# Central intra-lesional iron deposits as a possible novel imaging marker at 7 Tesla MRI in Susac Syndrome - an exploratory study

**DOI:** 10.1186/s12880-023-01171-7

**Published:** 2024-01-02

**Authors:** Daniel Strunk, Tim Sinnecker, Ilka Kleffner, Jan Doerr, Marius Ringelstein, Catharina C. Gross, Cornelius Deuschl, Stefan Maderwald, Harald H. Quick, Elif Yamac, Karsten H. Wrede, Markus Kraemer

**Affiliations:** 1 Department of Neurology, Alfried Krupp Hospital, Essen, Germany; 2 Medical Image Analysis Center (MIAC AG), Basel, Switzerland; 3 Department of Neurology, University Hospital Basel, Basel, Switzerland; 4 Department of Neurology, University Hospital Knappschaftskrankenhaus, Ruhr University Bochum, Bochum, Germany; 5 Department of Neurology, Oberhavel Kliniken, Hennigsdorf, Germany; 6 Max Delbrueck Center for Molecular Medicine and Charité – Universitätsmedizin Berlin, corporate member of Freie Universität Berlin, Humboldt-Universität zu Berlin, Berlin Institute of Health, Berlin, Germany; 7 Department of Neurology, Medical Faculty, Heinrich-Heine University Düsseldorf, Düsseldorf, Germany; 8 Department of Neurology, Center for Neurology and Neuropsychiatry, LVR-Klinikum, Heinrich-Heine-University Düsseldorf, Düsseldorf, Germany; 9 Department of Neurology with Institute of Translational Neurology, University Hospital Münster, Westfälische Wilhelms University of Münster, Münster, Germany; 10 Department of Diagnostic and Interventional Radiology and Neuroradiology, University Hospital Essen, University Duisburg-Essen, Essen, Germany; 11 Erwin L. Hahn Institute for Magnetic Resonance ImagingEssen, Germany & High Field and Hybrid MR Imaging, University Duisburg-EssenUniversity Hospital Essen, Essen, Germany; 12 Department of Intracranial Endovascular Therapy, Alfried Krupp Hospital, Essen, Germany; 13 Department of Neurosurgery and Spine Surgery, University Hospital Essen, 45147 Essen, Germany; 14 Department of Neurology, University Hospital Giessen and Marburg, Marburg, Germany

**Keywords:** Susac syndrome, 7 Tesla MRI, Central vein sign, T1 hypointense lesions, “Iron dot” lesions, Imaging marker

## Abstract

**Background:**

Susac syndrome (SuS) is a rare autoimmune disease that leads to hearing impairment, visual field deficits, and encephalopathy due to an occlusion of precapillary arterioles in the brain, retina, and inner ear. Given the potentially disastrous outcome and difficulties in distinguishing SuS from its differential diagnoses, such as multiple sclerosis (MS), our exploratory study aimed at identifying potential new SuS-specific neuroimaging markers.

**Methods:**

Seven patients with a definite diagnosis of SuS underwent magnetic resonance imaging (MRI) at 7 Tesla (7T), including T2* weighted and quantitative susceptibility mapping (QSM) sequences. T2 weighted hyperintense lesions were analyzed with regard to number, volume, localization, central vein sign, T1 hypointensity, and focal iron deposits in the center of SuS lesions (“iron dots”). Seven T MRI datasets from the same institute, comprising 75 patients with, among others, MS, served as controls.

**Results:**

The “iron dot” sign was present in 71.4% (5/7) of the SuS patients, compared to 0% in our control cohort. Thus, sensitivity was 71.4% and specificity 100%. A central vein sign was only incidentally detected.

**Conclusion:**

We are the first to demonstrate this type of “iron dot” lesions on highly resolving 7T T2*w and QSM images in vivo as a promising neuroimaging marker of SuS, corroborating previous histopathological ex vivo findings.

**Supplementary Information:**

The online version contains supplementary material available at 10.1186/s12880-023-01171-7.

## Background

Susac syndrome (SuS) is a rare CD8 T-cell mediated endotheliopathy characterized by a clinical triad of encephalopathy, sensorineural hearing loss, and visual impairment due to branch retinal artery occlusions (BRAO) [1, 2]. The diagnosis of SuS is often hampered by the fact that the complete clinical triad rarely presents at disease onset and clinical presentation often overlaps with differential diagnoses, such as multiple sclerosis (MS). Thus, a comprehensive diagnostic workup, including (i) brain magnetic resonance imaging (MRI), (ii) audiogram, and (iii) fluorescein angiography (FA) is crucial [3].

Characteristic MRI findings of SuS comprise “snowball” like lesions in the central part of the corpus callosum. Grey matter lesions, leptomeningeal enhancement, microinfarcts of the thalamus and internal capsule, and cerebellar lesions are less frequent [4]. More advanced MRI techniques such as Diffusion Tensor Imaging (DTI) and highly resolving gradient echo T2* weighted (T2*w) imaging at 7 Tesla (T) were previously shown to underline the destructive nature of the disease and its lesions [5–7]. We have previously demonstrated that brain lesions in SuS patients, in contrast to MS patients, are typically lacking a central vein sign (CVS) [7, 8]. Recently, central nervous system (CNS) histopathology of seven SuS patients revealed that accumulation of CD8 T cells in brain microvessels is associated with microhemorrhages and iron deposition around blood vessels [2]. To investigate whether MRI can visualize perivascular iron deposits in vivo, we performed 7T ultrahigh field MRI in a cohort of seven SuS patients. Results were compared to a previously published control group with MS, Baló’s concentric sclerosis or cerebral aneurysms [9–14].

## Methods

### Study design and participants

For this ultrahigh field MRI study, seven patients with a definite diagnosis of SuS, according to the European Susac Syndrome Consortium (EUSAC), were recruited at a German center on the 28th of February in 2020 during an information event for SUS patients [3]. Exclusion criteria were the inability to undergo a 7T MRI examination, including but not restricted to cochlea implants, tattoos, dental implants, history of seizures, pregnancy, and the inability to provide informed consent. All subjects were examined shortly after inclusion in the study.

The local ethics committee of the University Duisburg-Essen approved the study (16-7214-BO). It was conducted in accordance with the Declaration of Helsinki in its currently applicable version, the guidelines of the International Conference on Harmonisation of Good Clinical Practice (ICH-GCP), and the applicable German laws. All participants gave informed written consent.

### MRI data acquisition

All ultrahigh field MR images were acquired at the Erwin L. Hahn Institute Essen, Germany, using a 7T whole-body MR system (Magnetom 7T, Siemens, Erlangen, Germany), equipped with a gradient system that provides a 40 mT/m maximum amplitude and a slew rate of 200 mT/m/ms. The imaging coil was a one-channel transmit/32-channel receive head radiofrequency coil (Nova Medical, Wilmington, USA). The imaging protocol included (i) a 3D T1-weighted magnetization-prepared rapid acquisition and multiple gradient echoes with 2 inversions (MP2RAGE; TE 2.9 ms; TR 5600 ms; TI1 1000 ms; TI2 2900 ms; acquisition time 12 min, spatial resolution 0.6 × 0.6 × 0.6 mm³) yielding quantitative T1 maps and T1 weighted images, (ii) 3D double inversion recovery (DIR; TE 198 ms; TR 11,000 ms; acquisition time 7 min, spatial resolution 1.0 × 1.0 × 1.0 mm³), and (iii) a 3D multi-echo gradient echo sequence (5 echos, TE 5–25 ms; TR 28 ms; acquisition time 10 min, spatial resolution 0.7 × 0.7 × 0.7 mm³) yielding T2* weighted (T2*w, TE 25ms) and quantitative susceptibility mapping (QSM) images.

### Image analysis and lesion characterization

All MRI data were processed using 3D Slicer (The Slicer Community, version 4.11). The total lesion load was determined on DIR images and confirmed on quantitative T1 maps by manually masking all lesions by two experienced investigators to finally calculate the numbers and volumes of lesions. A white matter lesion was defined as a T2 DIR hyperintensity, extending over at least five voxels. Virchow Robin spaces were excluded by their DIR hypointense signal and tubular appearance. We differentiated periventricular lesions, juxtacortical lesions, subcortical lesions, cortical lesions, lesions within the corpus callosum, and other white matter lesions that did not fullfill the criteria of the aforementioned locations.

The existence of a central vein was assessed on T2*w images. According to the North American Imaging in Multiple Sclerosis (NAIMS) criteria, a central vein was identified as a hypointense linear structure running through the center of a lesion in equidistance to its borders [15].

Lesion masks were co-registered by using affine registration to T1 maps calculated from MP2RAGE data as shown previously to assess intralesional T1 [16].

Finally, intralesional iron deposition was visually assessed on T2*w images and QSM and defined as strongly T2*w hypointense, punctate paramagnetic signal alterations showing high magnetic susceptibility on QSM. For comparison, we reanalyzed the presence of positive “iron dot” sign in MRI datasets from 75 previously published patients (52 with cerebral aneurysms, 12 with MS as defined by the McDonald criteria, 10 with Baló’s concentric sclerosis without clinical signs of MS, and one with MS and concomitant progressive multifocal leukoencephalopathy) examined in the same institute [9–14]. This selection comprised the most suitable control subjects, examined with the same scanner. Clinical information were neither available to the performers of 7T MRI nor to the radiologists who evaluated the images. Analysis of cerebral MRI scans was conducted by two independent raters (TS, EY). Discrepancies in rating results were resolved by consensus after further analysis of the respective MRI scans.

## Results

### Cohort description

The cohort under investigation comprises seven patients (five females, mean age 37.4 ± 6.96 years at the time of MRI, see Table 1). All patients were diagnosed with a definite SuS according to the EUSAC criteria, i.e. presented with the triad of encephalopathy, sensorineural hearing loss, and visual disturbances due to BRAO. Disease status was classified as ‘stable’ or ‘in remission’ in all patients, when MRI was acquired. Only two patients suffered from concomitant diseases, partially of inflammatory origins, such as Hashimoto’s thyroiditis and pernicious anemia (see Table 1). All patients had a history of glucocorticoid treatment, whereas, at the time of MRI, only one female patient was still treated with intravenous immunoglobulins (IVIG) as maintenance therapy. Apart from that, varying therapeutic regimens were administered after glucocorticoids during the active stage of the disease, with IVIG being the second and rituximab being the third most common treatment (see Table 1). There are no records of any adverse events due to 7T MRI in this study.

**Table 1:** Demographic and clinical characteristics of included patients with Susac Syndrome

Demographic characteristics						Clinical signs of Susac syndrome					Therapy***			
Patient	Sex [m = male; f = female]	Age at disease onset	Age at diagnosis	Age at MRI**	Encephalopathy [yes = 1; no = 0]		BRAO* [yes = 1; no = 0]	Labyrinthine deafness [yes = 1; no = 0]	Disease activity at MRI** [active = 1; stable = 0]	Comorbidities		Past	Current [0 = none]	
#1	m	32	33	40	1		1	1	0	-		MP, PLEX, CYC, RTX, AZA	0	
#2	f	32	34	38	1		1	1	0	-		MP, IVIG	0	
#3	m	24	26	42	1		1	1	0	vitiligo, pernicious anemia, lichen sclerosus, chronic bronchitis, pyelonephritis		MP	0	
#4	f	29	30	37	1		1	1	0	-		MP, IVIG, RTX	IVIG	
#5	f	18	18	22	1		1	1	0	-		MP, IVIG	0	
#6	f	32	33	37	1		1	1	0	-		RTX	0	
#7	f	34	35	46	1		1	1	0	Hashimoto’s thyroiditis		MP, IVIG	0	

* Branch retinal arterial occlusions; ** Magnetic resonance imaging; ***, AZA = Azathioprine, CYC = Cyclophosphamide, IVIG = Intravenous immunoglobulins,

M*p* = Methylprednisolone, PLEX = Plasmapheresis, RTX = Rituximab

### Burden and localization of lesions in SuS patients

Interindividual lesion load varied strongly. The mean lesion number was 22.57 ± 9.97 and the mean lesion volume was 20.6 ± 54.19 mm³ (see Table 2). The total number of detected lesions in all patients was 193. Analyzing the localization of the lesions yielded a numerical predominance of ‘other white matter lesions’, followed by callosal, periventricular, and subcortical lesions. We did not observe (juxta-) cortical lesions (see Table 2). We did not identify any infratentorial lesions.

**Table 2:** Burden and localization of T2/Fluid attenuated inversion recovery (FLAIR) hyperintense lesions on 7 Tesla Magnetic Resonance Imaging

	Mean	Median	Range	Standard Deviation
Number of lesions	22.57	26	16–42	9.97
Lesion volume [mm^3^]	20.6	6.69	0.43–442.15	54.19
Lesion size [voxel]	95.37	31	2–2047	250.86
Localization of lesions	Total number	Percentage of all lesions [%]		
- Corpus callosum	56	29.02		
- Periventricular	17	8.81		
- Juxtacortical	0	0		
- Other white matter lesions	103	53.37		
- Cortical	0	0		
- Subcortical - Overall	17 193	8.81 100		

### “Iron dot” lesions

**Fig. 1:**
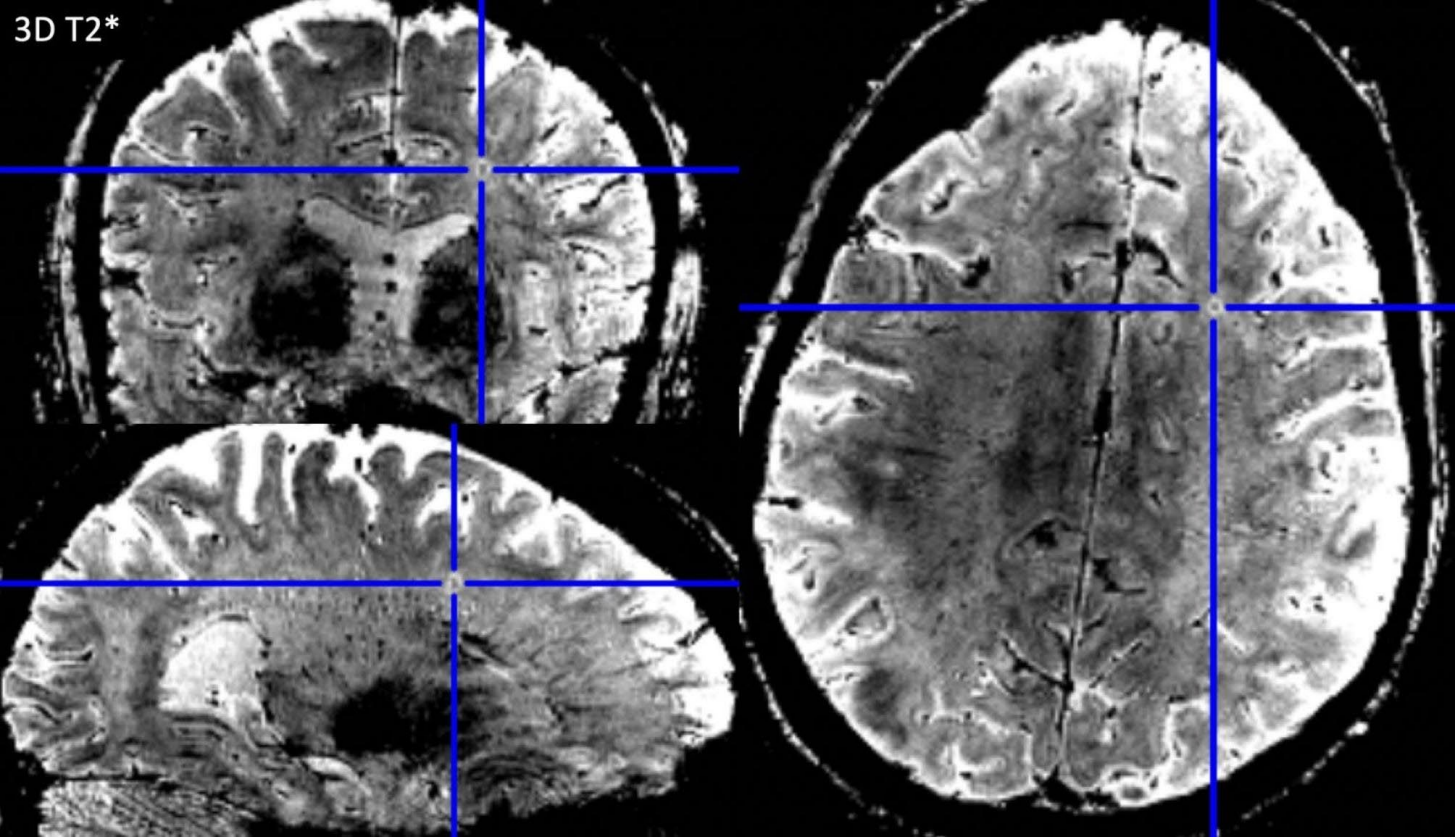
“Iron dot” lesion in the left frontal lobe on 3D T2* weighted coronal (top left), sagittal (lower left), and axial (right) 7 Tesla magnetic resonance imaging sequences. Please note the strong but punctate and sharply delineated intra-lesional signal loss on T2*w. This type of intra-lesional T2*w signal loss differs from that of more recent multiple sclerosis (MS) lesions where central T2*w hypointensities appear larger and more diffuse. They are also distinct from (enlarged) venules present in MS lesions as “iron dot” lesions do not show a tubular appearance following the course of a vessel

**Fig. 2:**
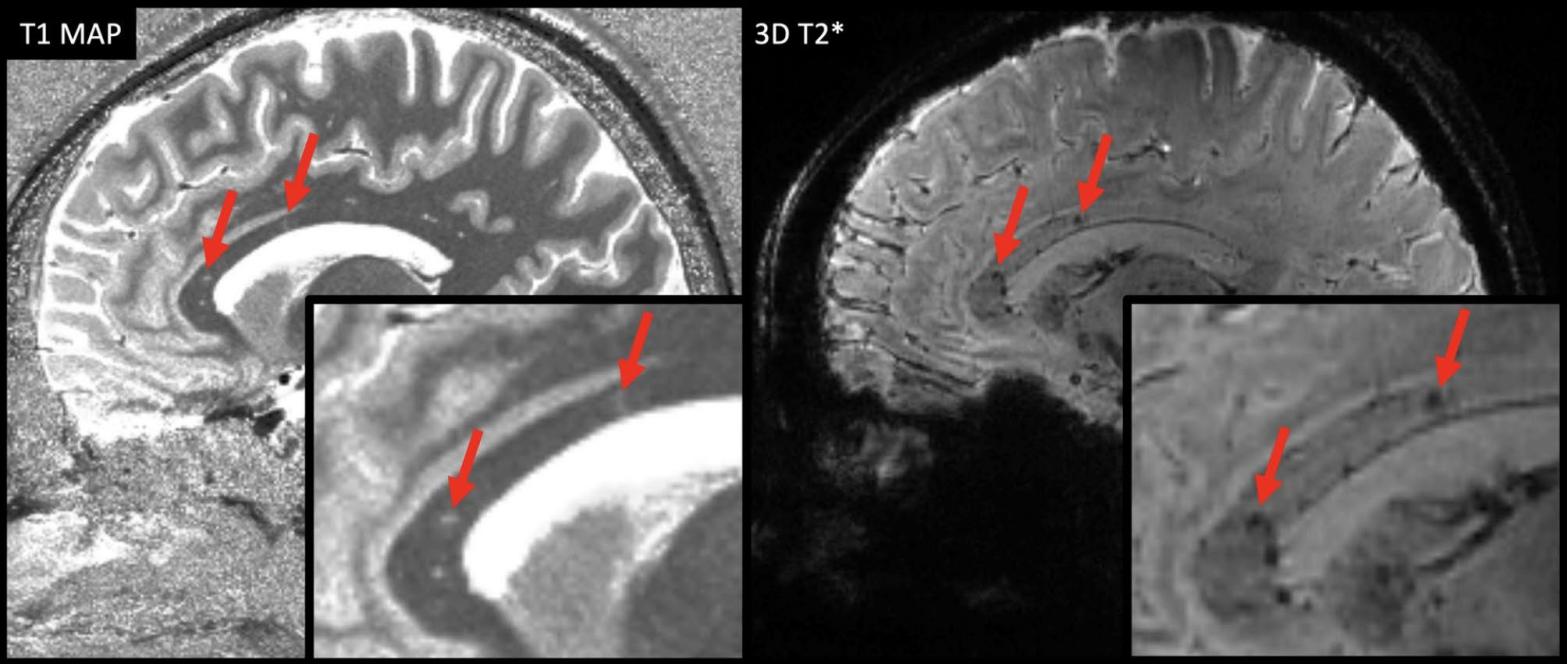
Callosal “iron dot” lesions (indicated by red arrows) on 7T quantitative T1 maps (left) and 3D T2* weighted (right) sequences

Of all 193 lesions, 11.4% were classified “iron dot” lesions (see Table 3). The “iron dot” sign was present in 71.4% (5/7) of the SuS patients, compared to 0% in our control cohort. Thus, sensitivity was 71.4% and specificity 100%. A median number of 2 (range: 0–9) iron dot lesions per patient was found in our cohort. This lesion type accounted for a median of 5.41% (range: 0-56.3%) of all cerebral lesions in an individual patient. While mean volume suggests a difference in lesion size in favor of non-iron lesions, the median volumes of iron versus non-iron lesions were comparable. Iron dot lesions were predominantly visualized in callosal and ‘other white matter lesions’ (see Table 3, Figs. 1 and 2, and Supplemental Fig. 1).

**Table 3:** Morphology and localisation of “Iron dot” lesions on 7 Tesla Magnetic Resonance Imaging

	Number of lesions	Percentage [%]	Mean volume [mm^3^]	Median volume [mm^3^]	Range volume [mm^3^]	Standard deviation volume [mm^3^]
Iron lesions	22	11.4	13.48	6.05	0.648–100.01	20.64
Non-iron lesions	171	88.6	21.52	6.7	0.43–442.15	57.02
Localisation of iron lesions						
- Callosal	7	31.82	8.98	3.46	0.65–29.38	9.97
- Periventricular	0					
- Juxtacortical	0					
- Other white matter lesions	14	63.64	16.46	8.53	0.86–100.01	24.36
- Cortical	0					
- Subcortical	1	4.55	3.24	3.24	3.24–3.24	0

### Central vein sign

In our cohort of SuS patients, we only incidentally detected a central vein sign (CVS), and the percentage of CVS-positive lesions, 4.5%, did not reach a threshold value of 40% or 50% that is considered to differentiate between MS and differential diagnoses in any patient [8, 17]. In the included patients with SuS, all lesions with CVS were classified as ‘other white matter lesions‘.

### T1 hypointense lesions

The visual analysis yielded 85 T1 hypointense and cerebrospinal fluid (CSF) isointense lesions, which accounted for 44% of all detected lesions on MRI scan and, for the most part, did not show any iron deposits (see Table 4). Most of these lesions were detected in the corpus callosum, followed by ‘other white matter lesions’, periventricular, and subcortical lesions (see Table 4).

**Table 4:** Visual and quantitative assessment of T1 hypointense lesions on 7 Tesla Magnetic Resonance Imaging

	Visual analysis		Quantitative analysis°			
	Number of T1 hypointense lesions	Percentage of total number of T1 hypointense lesions [%]	Mean	Median	Standard deviation	Range
**All lesions**	85	100	2434.02	2402.27	194.34	2095,8-3256,04
Iron lesions	10	11.76	2433.57	2404.5	234.91	2133–3256,04
Non-iron lesions	75	88.24	2434.08	2402.27	188.49	2095,8-3057,31
Callosal	36	42.35	2489.93	2482.86	214.91	2133–3057,31
Periventricular	8	9.41	2526.51	2555.12	133.48	2271,58-2757,38
Juxtacortical	0	0	-	-	-	-
Other white matter lesions	32	37.65	2395.99	2366.89	182.63	2117,6-3256,04
Cortical	0	0	-	-	-	-
Subcortical	9	10.59	2387.79	2453.07	164.31	2095,8-2611,47
Large lesions*	55	100	2508.93	2486.61	192.83	2168.91–3256.04
Iron lesions	7	12.73	2566.96	2514.93	253.83	2260.62–3256.04
Non-iron lesions	48	87.27	2502.03	2484.67	183.01	2168.91–3057.31
Callosal	18	32.73	2632.81	2655.85	209.28	2190.57–3057.31
Periventricular	6	10.91	2529.3	2534.27	133.85	2271.58–2757.38
Juxtacortical	0	0	-	-	-	-
Other white matter lesions	26	47.27	2462.18	2427.33	175.79	2168.91–3256.04
Cortical	0	0	-	-	-	-
Subcortical	5	9.09	2420.33	2461.26	143.51	2170.39–2611.47
Small lesions*	28	100	2360.23	2316.62	167.81	2095.8–2999.83
Iron lesions	3	10.71	2322.41	2312.9	142.47	2133–2631.37
Non-iron lesions	25	89.29	2365.63	2316.62	170.44	2095.8–2999.83
Callosal	16	57.14	2383.27	2357.71	153.76	2133–2817.31
Periventricular	2	7.14	2519.81	2555.12	132.36	2292.44–2696.26
Juxtacortical	0	0	-	-	-	-
Other white matter lesions	6	21.43	2330.81	2301.7	166.66	2117.6–2999.83
Cortical	0	0	-	-	-	-
Subcortical	4	14.29	2358.87	2299.4	175.83	2095.8–2586.21

° Quantitative analysis was performed by using T1mean, i.e. mean T1 time for all voxels of the respective lesion

*Large/small lesions were larger or smaller than the median lesion size of 6.69 mm^3^

In addition to this visual analysis, we performed a quantitative assessment of intra-lesional T1 on quantitative T1 maps. The mean intra-lesional T1 time for all voxels of the respective lesions was not significantly longer in periventricular and callosal lesions compared to subcortical or other white matter lesions. The mean T1 was significantly longer in large versus small lesions (*p* < 0.01) (see Table 4). This relationship, however, might be biased by proportionally larger partial volume effects in small versus large lesions.

## Discussion

Our study used an advanced MRI protocol at 7 T to identify potential new imaging markers in SuS. We investigated whether MRI can visualize perivascular iron deposits *in vivo.* Hereby, we are the first to demonstrate signs of focal iron deposits in the center of a subgroup of SuS lesions (“iron dots”) in most of the included patients. Up to the present, microhemorrhages in close proximity to damaged small vessels with endothelial cell injury in SuS patients were exclusively reported ex vivo [2]. Taking the opportunities of the improved sensitivity of 7T MRI in detecting even smallest changes in magnetic susceptibility, our exploratory study is the first one to show the presence of small punctate spots of increased magnetic susceptibility within the center of SuS lesions on highly resolving 7T T2*w and QSM images in vivo [18]. In comparison with a control group of 75 patients with lesions of presumed vascular origin and neuroinflammatory lesions, who were examined at the same institute as our current cohort, the finding of “iron dots” in SuS patients turned out to be highly specific. In more detail, we analyzed 7T MRI scans of 52 patients with cerebral aneurysms, 12 with clinically definite MS, 10 with Baló’s concentric sclerosis, and one with MS and concomitant progressive multifocal leukoencephalopathy without identifying any “iron dots” [9–14]. Moreover, to the best of our knowledge, “iron dot” lesions were not described in relevant previous studies in other neuroinflammatory diseases, such as neuromyelitis optica spectrum disorder or in other inflammatory and non-inflammatory differential diagnoses [19]. Consequently, “iron dots” bear the potential to serve as a novel neuroimaging marker of SuS, if confirmed in a larger cohort.

Of note, there are similar MRI phenomena in other conditions, but these can usually be clearly distinguished from our findings.

First of all, susceptibility weighted imaging (SWI) hypointense signals can also be observed within MS lesions. SWI hypointense signals in MS usually relate either to (partially dilated) veins or are a result of demyelination and/or iron deposition. Especially in chronic, larger, and clearly T2w hyperintense lesions, the veins may be characterized by a very strong hypointense signal in comparison to the surrounding T2w shine through signal [20]. We have also described this phenomenon within highly destructive Balo lesions [10]. Veins can be identified by their partly tubular course within the lesion, especially when the lesion is viewed in all three planes. Besides dilated veins, microhemorrhages could also be an explanation for SWI hypointense signals. The latter could be distinguished from the central punctate hypointensities we observed in SuS by their non-central localisation within the lesion and their multiple occurrences in a lesion.

**Fig. 3:**
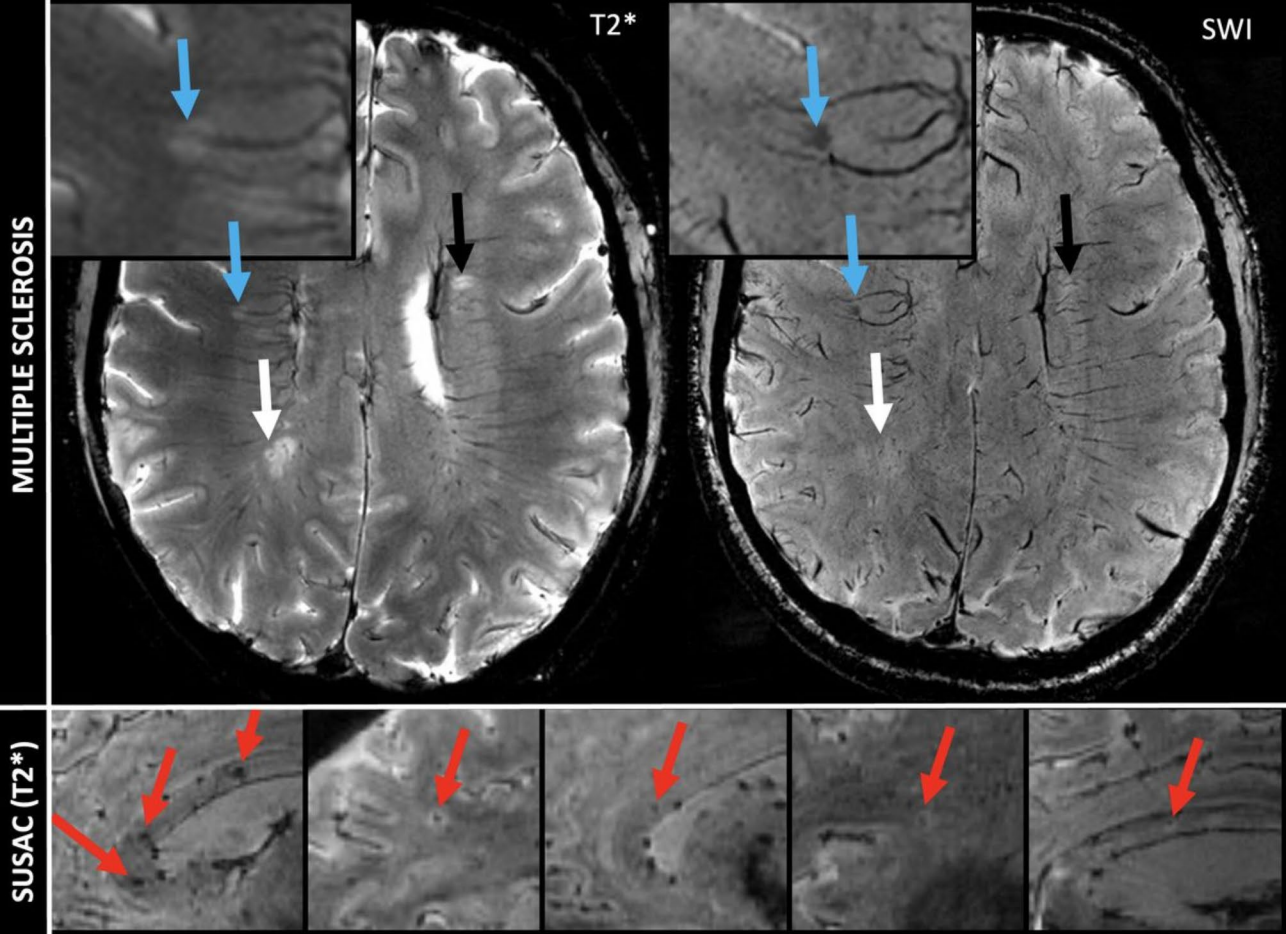
Comparison of lesion morphology of “iron dot” lesions in Susac syndrome (bottom) and multiple sclerosis (top). The figure illustrates differences in morphology on 7T T2*w images between “iron dots” in Susac syndrome (red arrows) and the central vein sign (white and black arrow) as well as T2*w hypointense core lesions (blue arrows with zoom) in multiple sclerosis (MS). All exemplary T2*w images in the bottom are from different Susac patients. Please note that the “iron dots” in Susac syndrome (red arrows) appear punctate and sharply delineated on T2*w. Contrarily, the T2*w hypointense core lesions (blue arrows with zoom) in MS often appear just slightly less hyperintense on T2*w in comparison to other MS lesions. Only when the lesion is viewed on strongly susceptibility weighted sequences (right) does a rather diffuse signal loss become apparent. The central vein sign in MS also appears as a point-like, pronounced hypointensity on T2*w images when the slice plane is perpendicular to the long axis of the MS lesion (white arrow). However, if the slice plane is parallel to the long axis of the lesion, the CVS can be recognized as a straight line running through the center of MS lesions (black arrow)

In contrast to microbleeds, the phenomenon described in our current study occurs strictly within otherwise T2w/Fluid attenuated inversion recovery (FLAIR) hyperintense lesions. Furthermore, by definition, microbleeds can reach a size of up to 10 mm. However, the changes we describe, are limited to a small spot. In this respect, we consider the changes in SuS to be morphologically different from microbleeds. Brain lesions in MS can also show paramagnetic signal changes in the center of rather new lesions as a result of demyelination or smaller iron deposits released by dying iron-rich oligodendrocytes. In this case, SWI hypointense signals are more diffusely distributed and occupy a larger proportion of the lesion, whereas we see a concentration of signal toward a small spot in Susac lesions. Also, “iron rims” can be observed around MS lesions as a potential imaging marker of iron-laden CD68 positive cells and chronic inflammation [20–22]. In the event of very small MS lesions, it is conceivable that similar appearances to those seen in SuS could occur. In this case, the distribution and localization of the lesions could help to differentiate between the two conditions. For differentiation between the “iron dot” sign in SuS and CVS and T2*w hypointense core lesions in MS see Fig. 3.

Strong signal loss on T2*w and high intensity on QSM suggest that ”iron dots” in SuS patients represent iron deposits rather than demyelination. A fragile blood-brain-barrier could well explain our observation of “iron dots” in SuS in the course of endothelial cell injury that enables the transit of iron into the central nervous system, resulting in focal iron deposits. These iron deposits could represent the equivalent to the microhemorrhages described in brain specimen of SUS patients, caused by endothelial damage. Signs of small vessel pathology in SuS were also observed with other advanced MRI techniques: Intracranial high-resolution vessel wall MRI was able to show inflammatory foci along small vessels, and black blood MRI - a technique sensitive to inflammatory changes within the vessel wall - delineated multiple foci of parenchymal contrast enhancement in a patient with SuS consistent with endothelial cell injury of small vessels [23, 24].

We also found that, in accordance with the literature, SuS lesions were often localized in the corpus callosum [4]. Despite using high resolution 7T images that are sensitive to cortical demyelination, (juxta-) cortical lesions were not observed.

In contrast to MS, lesions in SuS were stronger T1 hypointense, and less frequently exhibited a central vein than reported previously in MS [7, 8, 25]. As already outlined in the results section, none of the included patients exceeded the cut-off of 40–50% of lesions with CVS, so that those lesions with CVS have to be labelled ‘incidental findings’ [15]. Our study hence strengthens previous data that CVS can be considered as a tool or diagnostic marker to facilitate the differentiation between MS and SuS [7, 8, 26].

T1 hypointense and CSF isointense lesions represent another imaging marker in SuS and may represent severe tissue destruction [7]. For quantitative assessment of T1 as a marker of tissue microstructural integrity, MP2RAGE sequences are appropriate and robust [27, 28]. On highly resolving 7T MP2RAGE images, we found especially callosal and periventricular lesions showing prolonged T1 values in SuS patients. Our findings are well in line with previous visual analyses of CSF T1 isointensity, where CSF T1 isointense lesions were less frequent in SuS versus MS [7]. Also, MS patients rather show lesions in the lateral areas of the corpus callosum with a lower degree of reduction in T1w signal intensity [7]. In turn, a central localization of callosal lesions with severely prolonged T1 would be a more typical feature of SuS.

Our study has strengths and limitations. A main strength is the reevaluation and extension of known MRI characteristics of SuS in a new dataset, using the best available technique. Albeit MRI in our control group with differential diagnoses of SuS could not be performed retroactively, using precisely the same MR tomograph, protocol and head coil as technical modifications were made in the meantime, all images were generated at the same institute and offer the same high resolving and strong T2*w contrast and susceptibility effects as the images acquired in SuS with only minor differences in resolution and other parameters. They hence have a high degree of comparability with regard to detecting the smallest focal changes in magnetic susceptibility. Therefore, we believe that the “iron dots” finding in SuS is valid and that the comparison with already published data and images from the same institute underlines its specificity and potential significance. Limitations comprise a relatively small cohort of clinically stable SuS patients with a definite diagnosis. Patients in an early stage of SuS or with an clinically active disease were not included. Furthermore, a histopathological validation of observed changes on QSM/T2*w would have been desirable. Our study shows that the iron dot sign is present in SuS at 7T, but, given the poor availability of 7T MRI in most medical institutions, it would be also clinically relevant to analyze the sensitivity at 3T or 1.5T magnets in detecting this sign.

In summary, “Iron dot” lesions may represent a novel imaging marker specific to SuS. Still, its sensitivity, specificity, and association with disease activity need to be elucidated in future studies in larger cohorts which also comprise clinically active patients and individuals in early stages of SuS.

### Electronic supplementary material

Below is the link to the electronic supplementary material.

Supplementary Material 1

Supplementary Material 2

## Data Availability

Study data can be requested from Markus Kraemer (markus.kraemer@krupp-krankenhaus.de).

## List of abbreviations

BRAO: Branch retinal artery occlusion
CD: Cluster of differentiation
CNS: Central nervous system
CSF: Cerebrospinal fluid
CVS: Central vein sign
DIR: Double inversion recovery
DTI: Diffusion Tensor Imaging
EUSAC: European Susac Syndrome Consortium
FA: Fluorescein angiography
FLAIR: Fluid attenuated inversion recovery
ICH-GCP: International Conference on Harmonisation of Good Clinical Practice
IVIG: Intravenous immunoglobulins
MP2RAGE: Magnetization-prepared rapid acquisition and multiple gradient echoes with 2 inversions
MRI: Magnetic resonance imaging
MS: Multiple Sclerosis
NAIMS criteria: North American Imaging in Multiple Sclerosis criteria
QSM: Quantitative susceptibility mapping
Seven T: Seven Tesla
SuS: Susac syndrome
SWI: Susceptibility weighted imaging
T2*w: T2* weighted
