# Supplementary figures and images for: Central intra-lesional iron deposits as a possible novel imaging marker at 7 Tesla MRI in Susac Syndrome - an exploratory study
Source: BMC Med Imaging. 2024 Jan 2;24:4. doi: 10.1186/s12880-023-01171-7 (PMC10759674; doi:10.1186/s12880-023-01171-7)

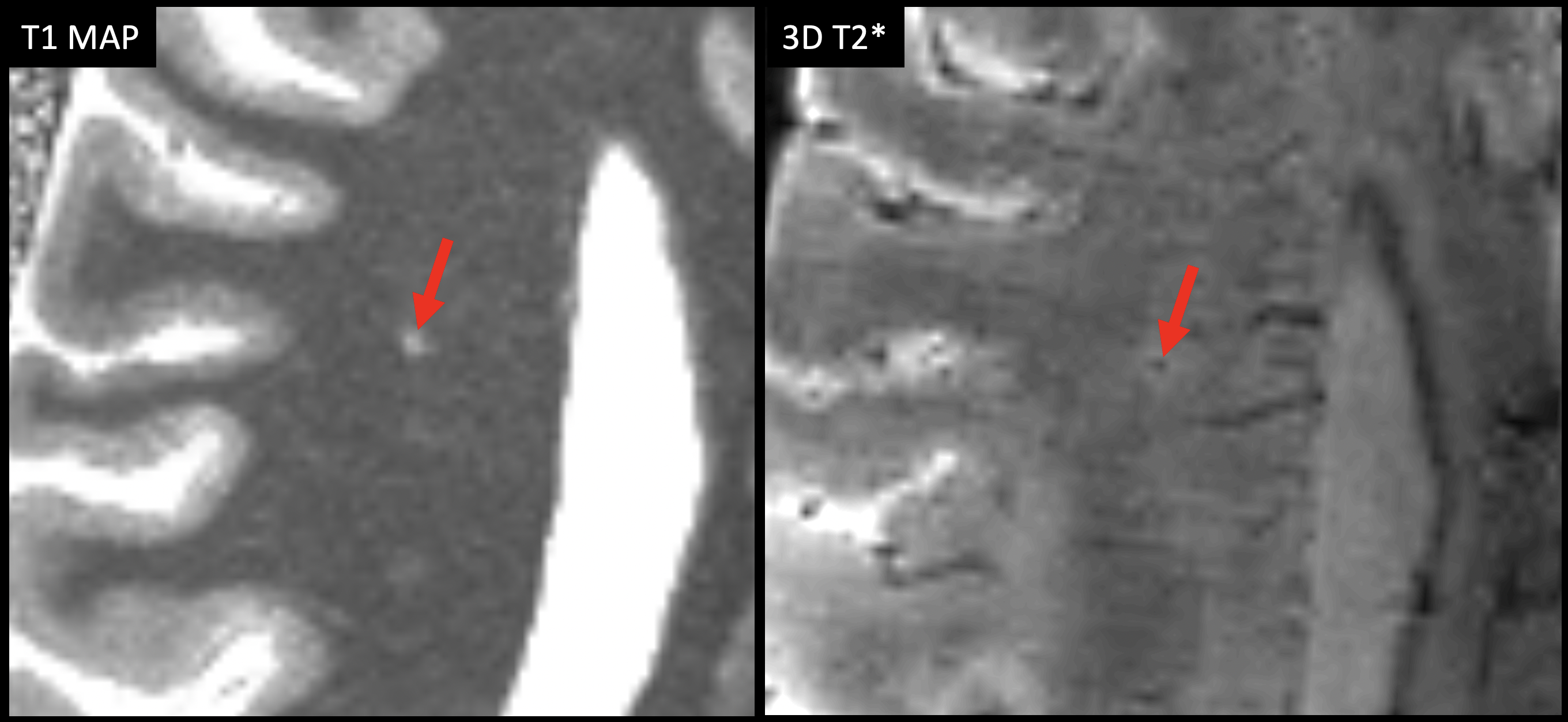

Supplement: Supplementary file 1 — Supplementary Material 1 [file 12880_2023_1171_MOESM1_ESM.png]
