# Supplementary material for: Central intra-lesional iron deposits as a possible novel imaging marker at 7 Tesla MRI in Susac Syndrome - an exploratory study
Source: BMC Med Imaging. 2024 Jan 2;24:4. doi: 10.1186/s12880-023-01171-7 (PMC10759674; doi:10.1186/s12880-023-01171-7)
Supplement: Supplementary file 2 — Supplementary Material 2 [file 12880_2023_1171_MOESM2_ESM.docx]

**Supplemental Figure 1.** Exemplary “iron dot” lesion (indicated by red arrow) on 7T quantitative T1 maps (left) and 3D T2* weighted (right) sequences.
